# JACMP 2015 – 2019

**DOI:** 10.1002/acm2.14452

**Published:** 2024-06-27

**Authors:** Per. H. Halvorsen

**Affiliations:** ^1^ Varian Advanced Oncology Solutions Newton Massachusetts USA

## 1 THE MATURE JACMP

The period 2015−2019 saw continued robust growth in submissions and published articles, with an average of 235 published articles per year compared to 137 in the prior 5‐year period, all while maintaining a manuscript acceptance rate near 50%. We successfully completed the transition to a new publisher (Wiley) in 2016, followed by a transition from 6 to 12 issues annually in 2019.

I'm fortunate to have had the opportunity to collaborate with Michael Mills and Tim Solberg over the past two decades and have learned so much from them about the evolution of our profession and the scientific publication models we've relied on to disseminate information. When Michael asked me to consider serving as Deputy Editor‐in‐Chief, I was grateful for the opportunity to contribute; however, modestly, to this effort. The Journal's success is the result of an incredible body of work by so many contributors, many of whom generously share their time and expertise in important but largely unseen ways. When you read a JACMP article, remember that its publication (and your free access to it) required significant time and effort by dozens of individuals.

The AAPM assumed responsibility for JACMP operations upon the decision in 2012 to have the AAPM assume all functions of the American College of Medical Physics. We continued using the Public Knowledge Project publishing platform and continued with the fully free business model (free to submit and publish and free to access), but the rapid growth of the journal inevitably ran up against basic financials—the higher volume of submissions meant significantly higher operating expenses. Michael Mills identified this dilemma in an editorial in 2015,^1^ and we implemented a $500 Article Publication Charge (APC) in November 2015. Though we could no longer continue with the fully‐free model, the APC was set significantly below the rate in similar journals, as explained in a subsequent editorial.^2^

Around the same time (mid‐2015), the AAPM formed an Ad Hoc Committee on Unifying Publication Platforms. As we stated in a 2016 editorial,^3^ it was no longer practical to continue publishing our two journals through two separate publishers using separate publication platforms, and the Public Knowledge Project platform used by JACMP (administered by Multimed) was no longer suitable for the current volume of submissions, advanced media requirements and large reviewer base. The Ad Hoc completed its work in July 2016, recommending that the AAPM contract with Wiley to publish both our journals. Given the stellar reputations of both journals and the impressive record of JACMP downloads (reaching 400,000 by 2017), the final AAPM‐Wiley agreement was very advantageous to our Association, with a net financial return exceeding $1 M annually. Wiley became the publisher of both journals in January 2017,^4^ followed by a concerted effort by the JACMP editorial board to ensure a smooth transition for our authors and readers, such as rebuilding our reviewer database among other “behind the scenes” priorities. The new publication platform and revenue models contributed to the JACMP returning a net positive margin to the AAPM in 2018 while publishing over 250 articles annually and seeing robust growth in downloads, reaching 640,000 in 2018 (from 400,000 in 2017).

The 2015−2019 time period also saw significant public policy initiatives aimed at moving more of the scientific publishing models toward open access. One of the more significant initiatives is Plan S, developed by a consortium of European research organizations. Michael Mills provided an excellent description of Plan S and the potential impact of similar initiatives in a 2019 editorial.^5^

Given the rapid growth in the number of articles processed each year, the Editorial Board made the decision in 2018 to transition from 6 to 12 issues annually, and this took effect with Volume 20 in 2019. With this change came a different “rhythm” as we had twice the number of deadlines and editorials, but the transition went very smoothly and has resulted in authors seeing their accepted article listed in an issue sooner.

Rather than list specific articles from the 2015 to 2019 time period, I'd like to summarize the focus and impact of published articles, as they align quite well with the most active clinical technology adoption challenges of this time period:

*2015 ‐ Volume 16*:

- Radiation Oncology: SBRT and new array and small‐field detectors; motion management, SGRT and 6DOF approaches for improved delivery accuracy; FFF dosimetry; automated population‐based planning.
- Imaging: 4DCT; dose reduction in CT; CBCT scatter correction; DECT.
- Management and Profession: MPPGs 3, 4 and 5.

*2016 ‐ Volume 17*:

- Radiation Oncology: Auto‐segmentation and automated planning; FFF dosimetry; single‐iso multi‐target SRS with VMAT; hypofractionation; protons vs. photon VMAT.
- Imaging: CT dose optimization.

*2017 ‐ Volume 18*:

- Radiation Oncology: Automation of planning workflows including the use of knowledge‐based planning (KBP) for dose prediction; scanned proton beams; small‐field modeling and measurement and SBRT delivery validation; MR‐guided therapy systems; 3D printing applications.
- Imaging: 4D MRI.
- Profession: MPPGs 6, 8 & 9.

*2018 ‐ Volume 19*:

- Radiation Oncology: KBP for automated planning and dose prediction; scanned proton beams; MR‐guided therapy systems; frameless CBCT/SGRT based radiosurgery; adaptive therapy.
- Imaging: Spectral CT, Digital Radiology QC.
- Profession: COMP CPQR guidelines; MPPG 10; impact of Artificial Intelligence.

*2019 ‐ Volume 20*:

- Radiation Oncology: Scanned carbon‐ion therapy and intensity‐modulated proton therapy; use of DECT in radiotherapy; using deep learning tools to improve MR‐only treatment planning; adaptive therapy; expanded 3D printing applications.
- Imaging: DECT, spectral CT.
- Profession: 3DCRT (3‐discipline Collaborative RT) special debates (medical physicist, radiation oncologist, and radiobiologist).

Looking at this trend in the focus and impact of published articles, I'm pleased to see that we largely succeeded in attracting and publishing articles that were highly relevant to the contemporary challenges faced by clinical medical physicists—fulfilling our mission as envisioned by every Editor‐in‐Chief since JACMP's founding, as succinctly stated by George Starkschall in 2008^6^: *“The real measure of impact of a paper in the Journal of Applied Clinical Medical Physics, then, is the number of times the information in that paper is used by a practitioner of medical physics to improve the quality of medical physics practice.”* By the close of 2019, by almost any metric (submissions, number and focus of published articles, downloads, and Journal Impact Factor), the JACMP had become a reliable venue for dissemination of timely and relevant clinical medical physics knowledge, to the benefit of every clinical physicist worldwide.
